# Laboratory and clinical teaching experience of nursing professors in the COVID-19 pandemic era: Now and the future

**DOI:** 10.3389/fpubh.2022.961443

**Published:** 2022-08-17

**Authors:** Seung-Yi Choi, Songxian Jin, Jung-Hee Kim

**Affiliations:** College of Nursing, The Catholic University of Korea, Seoul, South Korea

**Keywords:** clinical practice, COVID-19 pandemic, focus group interviews, laboratory practice, nursing faculty

## Abstract

Nursing professors must constantly interact with students, maintain a high level of professional performance, and meet targets and deadlines, even during a pandemic. Considering the changing educational environment, it is essential to identify contemporary limitations and problems to provide feedback for improvement. This study aimed to explore the laboratory and clinical teaching experiences of nursing professors during the COVID-19 pandemic. Focus group interviews were conducted with professors from the nursing departments of universities in Korea. In total, 19 professors who had laboratory and clinical experience participated in this study. The collected data were analyzed using thematic analysis. The analysis identified four themes. The themes included feeling helpless in the infection management system, uncertainty about the effectiveness of alternative practice training, acceptance of changes, and preparation for future practice training. As the necessity and possibility of non-face-to-face education have been confirmed by the pandemic, it is expected that classes using technology will be actively developed in nursing practice education. The roles and attitudes of teachers and educational institutions also need to change. Nursing professors should reflect upon and evaluate challenges to prepare for post-pandemic practical education.

## Introduction

The coronavirus disease 2019 (COVID-19) outbreak has adversely affected all social sectors worldwide, including the economy, healthcare, and education (1–5). Its high infectivity has necessitated measures such as social distancing, isolation, and travel restrictions, bringing almost all face-to-face activities to a standstill (1, 4, 6). In particular, the education system has been strongly affected by the COVID-19 pandemic (3, 7, 8). Schools and universities stopped all face-to-face classes, declared temporary closures, and struggled to find solutions (3, 8). To solve this problem, educational institutions worldwide switched to non-face-to-face remote classes, using online technology to maintain the learning process and achieve learning goals (3, 7, 9, 10).

Non-face-to-face classes have existed since before (11). Educational technology has advanced dramatically in the last few decades, and it has proven to be extremely useful during this pandemic (3). Online education is not a new concept for educators (11), and there are several online platforms that support online education (12). Nonetheless, universities have found it difficult to map their educational activities into the online space (7). Regardless of how advanced online learning becomes, it will never be able to replace the real classroom experience, the sacred teacher-student relationships, and the fun of studying in a real class with our peers (13). Furthermore, some students lack computers or mobile devices to study online learning and lack internet quotas (14). An unstable internet connection during lectures may limit classroom participation by making it difficult for teachers to interact with their students (15, 16). Medicine, nursing, veterinary medicine, agriculture, and engineering study programs, in particular, cannot be taught entirely online due to the extensive need for laboratory and internship experience (17).

Nursing education includes various clinical classes, and nursing students must complete a certain number of clinical hours (18). Nursing students in Korea are required to complete more than 1,000 h of clinical time during their enrollment in order to be placed on professional nursing staff after graduation (19). Overall, clinical practice represents an application-based curriculum that establishes the professional intuition of a nurse (20). While clinical practice should be conducted in the clinical field, many departments of nursing have stopped clinical practice due to the pandemic; thus, it is necessary to find countermeasures to prevent infectious diseases during clinical operations (21).

Furthermore, the reputation of educational units is increasingly under scrutiny. How well do they maintain their quality of education amidst this crisis by showing their adapting capabilities? Indeed, academic institutions are not able to transform all their college curricula into online resources promptly (3). University professors must constantly interact with students, maintain a high level of professional performance, and meet targets and deadlines even during a pandemic (22). Both professors and students at universities have experienced non-face-to-face classes for the first time. However, professors are obliged to choose the best teaching methods to provide students with a high-quality education. In addition, professors can use a combination of audio, video, and text to reach out to their students during this time of crisis to maintain a human touch (3).

The professor should choose various teaching methods suitable for nursing education, integrate students' active learning, and establish strategies to promote critical thinking while also playing the role of facilitator, so that students can integrate theory into clinical practice (23). Education includes more than just delivering knowledge through lectures and teaching students in clinical practice. Regardless of the physical and temporal distance from students, professors should help and support students in building their knowledge (24).

Previous studies related to the experience of clinical education during the pandemic have mostly focused on the students (20, 25, 26). Considering the changing educational environment, it is essential to identify contemporary limitations and problems to provide feedback for its improvement. For future innovation in laboratory and clinical education, it is necessary to understand the experience of the professor's education in this chaotic era.

Focus group interviews are in-depth interviews that involve posing open-ended questions and follow-up probes designed to obtain an in-depth understanding of participants' experiences, perceptions, opinions, and feelings. In focus groups, emphasis is placed on group interactions by encouraging participants to talk with each other, ask questions, and comment on experiences and personal perspectives (27). The focus group interviews can clarify the experience of nursing professors in the laboratory and clinical teaching. Thus, this study aimed to identify the experiences of nursing professors' laboratory and clinical teaching in the pandemic era and reflect on the future of nursing education.

## Methods

### Design

This qualitative study applied the thematic analysis method of Braun and Clarke (28) to explore the laboratory and clinical teaching experiences of nursing professors during the COVID-19 pandemic. This study followed the Consolidated Criteria for Reporting Qualitative Studies, which covers the reporting of studies using interviews and focus groups (29).

### Participants

The study participants were nursing professors who had experience in teaching laboratories and clinical experience. The inclusion criteria were tenured, teaching for at least 3 years, and having operated from 2020 to the present. The exclusion criteria were sabbatical or research years and no classes for more than one semester since 2020. Participants were recruited by posting and promoting online announcements to members of the Korean Society for Simulation in Nursing (KSSN).

An ideal focus group interview sample should include 5–8 participants; in groups larger than 10, participants find it difficult to freely share their views and thoughts. The most common and comfortable groups have 5–6 participants (27). However, because the scope of experience was simple and limited, this study decided to include 20 participants, five in each group.

Twenty professors voluntarily applied for participation, but one person withdrew; thus, 19 people participated. All participants were female in their 30 and 50s, and their educational careers ranged from at least 3 years and 6 months to 16 years and 3 months. Their subjects included fundamentals of nursing practice, adult nursing practice, pediatric nursing practice, women's health practice, psychiatric nursing practice, community nursing practice, and simulation practice.

### Ethics

This study was approved by the C University Institutional Review Board (IRB NO.2021-1445-0001). The participants were assured of confidentiality and informed that participation was voluntary, and that they could withdraw from the study at any time. They were informed that the transcripts would not be used for any purpose other than research.

### Data collection

Data were collected *via* focus group interviews in July 2021. Nursing professors who understood the purpose of this study and voluntarily signed the consent form participated in the study. The focus group interviews consisted of 4–6 participants each, and each group was interviewed once for ~60–90 min. The data were processed until saturation. Furthermore, when additional information was required, a telephone or message was used. Interviews were conducted in places where participants were comfortable with online, non-face-to-face interviews. The interviews were recorded after obtaining the consent of the participants. During the interview, all participants were allowed to use a pseudonym or freely turn the camera on and off. The burden of the interviews was minimized, and the participants were allowed to talk freely. The interviews were conducted by two female researchers who had previously conducted several focus group interviews and had practical lecture experience as a nursing professor and a doctoral student. The questions were composed of opening, introductory, transition, key, and ending questions. The key questions were as follows: “What was your experience in laboratory and clinical teaching in the pandemic era?” and “How does it compare to before?”

### Data analysis

The recorded interview content was transcribed and analyzed using thematic analysis, as described by Braun and Clarke (28). Thematic analysis is a useful method for analyzing the perspectives of subjects; it is not judged by the frequency of occurrence of words, and it is a flexible analysis that requires the analysis of potential meaning rather than the meaning of superficial content (30). The thematic analysis consisted of six phases. The initial phase of thematic analysis aimed to familiarize researchers with the collected data. Repeated reading contributed to better understanding and enhanced the researchers' familiarity with the data. Following the initial stage, initial codes were generated from the dataset that had a reoccurring pattern in the second phase. Coding was carried out through a systematic way of organizing and obtaining meaningful characteristics of the data related to the research question. Two researchers independently conducted the coding. The third phase, searching for themes, focused on a broader level of analysis and involved the researchers identifying suitable themes to which codes could be attributed. More specifically, initial codes pertinent to the research question were integrated into themes, considering how relationships were formed between codes and potential themes. In the fourth phase, the relationship between codes that could be included in the identified topic and other topics or codes was investigated; the similarities and differences were also classified and analyzed. The content of the extracted coding data was fully included in each subtheme by reviewing the formed themes. The identification possibility, interrelationship, and amount of code were considered using the recombination and separation method of the code. Consistency and clear patterns were also confirmed. In five phases, defining and naming themes were completed by refining the existing themes and subthemes presented in the final analysis. The final phase included writing a report that maintained consistency and logic and accurately described the results. In this process, after consulting with two qualitative research experts, each distinctive theme was defined.

### Rigor

To guarantee the rigor of the research, we endeavored to have credibility, transferability, dependability, and confirmability, as suggested by Lincoln and Guba (31), at each stage of the thematic analysis.

## Results

As a result of the analysis, 48 subcategories were generated, and nine categories were constructed by classifying subcategories related to each other. Further themes were derived from the four principal themes: (1) feeling helpless in the infection management system, (2) uncertainty about the effectiveness of alternative practice training, (3) acceptance of changes, and (4) preparation for future practice training (Table 1).

**Table 1:** Summary of themes, categories, and sub-categories.

**Themes**	**Categories**	**Subcategories**
Feeling helpless in the infection management system	Unpredictable situations	Inconsistent infection control guidelines
		Difficulty in decision making
		Sudden notice
		Persistence of tense situations
		Cessation of practice
		Inevitable situation
	Limitations of class management	Concerns about infection
		Students' anxiety
		Practice operations where maintaining distance is difficult
		Lethargy
		Increasing class hours
		Burnout
		Resignation
Uncertainty about the effectiveness of alternative practice training	Lack of educational resources	Lack of support from schools
		Lack of educational contents
		Shortage of faculty
		Difficulty finding educational methods
		Readjustment of evaluation methods
	Concerns about securing the quality of practice	Concerns about not being equivalent to clinical practice
		Concerns about students graduating without practice
		Insufficient time for clinical practice
		Concerns about students' diminished practical skills
		Guilt about students
	Negative feedback from students	Low lecture evaluation scores
		Non-face-to-face training is not recognized as practice
		Limitations of non-face-to-face practice
		Dissatisfaction with the operation of non-face-to-face practice training
		Dissatisfaction with the amount of assignments
Acceptance of changes	Reconsideration of traditional practice methods	Students accustomed to alternative practice
		Need for clinical practice appropriate for course characteristics
		Need for a flexible practice operation method
		Limitations of traditional clinical practice
	Confirmation of the feasibility of new practice training methods	Increased students' interest
		Improvement of students' self-confidence
		Safe educational environment
		Possible repeated learning
		Accumulation of know-how
		Expanded technical support
		Diversity of contents
Preparation for future practice training	Development of technology-based curriculum for practice	Learners as digital natives
		Joining the new normal era
		Development of practice training tailored to the characteristics of Generation Z
	Responding to the changing practice training environment	Exchange of information on new practice methods
		Clinical advice through industry-university cooperation
		Need to build a network for developing educational contents

### Feeling helpless in the infection management system

The participants were placed in an unpredictable situation, where their practice was stopped due to inconsistent infection control guidelines and the continuation of tensions. They expressed the limitations of class operation due to concerns about infection, students' anxiety, and difficult practice operations to maintain distance.

#### Unpredictable situations

The participants had difficulty in making decisions on the clinical and laboratory practice plans due to inconsistent infection control guidelines. They expressed that the inevitable and tense situations continued, such as receiving sudden notice from school and government.

“Because the guidelines of the country and the Ministry of Education are constantly changing, I think the practice class schedule has been adjusted around 15 times. Everyday things become uncommon, and my plans to do better become meaningless....” (participant 10).

“Even before the start of the class, I wondered whether students would go to clinical practice or not, but I got flustered when I heard that the practice was completely stopped just 1 week before the class started” (participant 15).

#### Limitations of class management

Participants were concerned about infection, students' anxiety, and practicing operations where it was difficult to maintain distancing. They felt helpless and were exhausted by the increased class hours required to prevent the spread of infectious diseases. They expressed their feelings of resignation regarding the inevitability of the situation.

“It has no problem when students sit and listen to the class. However, when students practice with dummies, they sometimes have to gather, so it is difficult to maintain distance due to the nature of the basic nursing practice. In such a case, we had no choice but to follow quarantine guidelines, such as wearing a mask and sanitizing hands, and continuing the class with the minimum number of students widening the distance from each other” (participant 12).

“First of all, it consumes a lot more energy than before... I had to make a separate program for a few people because I was told that 'Do not force students to participate in the practice' or 'Do not create a situation where students are anxious and unable to speak'. It was very difficult” (participant 10).

### Uncertainty about the effectiveness of alternative practice training

Participants tried to operate it as an alternative practice training in an unexpected situation; thus, it was difficult to find alternative education methods due to lack of support from schools and educational content, shortage of faculty, and difficulty in the readjustment of evaluation methods. In addition, participants were concerned about the deterioration of their skills because alternative training was not equivalent to clinical practice and graduation without clinical practice. In particular, there was uncertainty about the effectiveness of alternative practice training with negative feedback from students who did not recognize low lecture evaluation scores and non-face-to-face training as practice.

#### Lack of educational resources

Participants faced difficulty finding alternative education methods due to lack of support from schools and educational content, shortage of faculty, and difficulty in the readjustment of evaluation methods.

“To shoot VOD, I needed support from the school for everything including the video system, but it's not like the school is spontaneously ready to support it; so, I had to look for various ways” (participant 1).

“It was very difficult because I was not prepared for a scenario that had to be improvised or for a non-face-to-face operation through ZOOM” (participant 9).

#### Concerns about securing the quality of practice

Participants were concerned about the curriculum not being equivalent to clinical practice and students graduating without clinical practice. When the laboratory practice was changed to online, they expressed concerns and guilt regarding students' diminished practical skills.

“The quality of practice cannot be taken into account. In any case, I had to control the students so that they could complete their practice time… also felt a great sense of disparity about the fact that students had to take the practical exam without experiencing real practice in practice training. I could not evaluate them in the same way as before” (participant 10).

“Is it really okay for students to graduate? Can they go to the clinic? What is the level of students' clinical understanding? Can I ignore it and move on? These remain areas of my greatest concern” (participant 17).

#### Negative feedback from students

Some participants experienced feelings of depression because of the low lecture evaluations received from students after non-face-to-face practice. Students expressed that they did not recognize the non-face-to-face practice as practice, and they were dissatisfied with the operation of the non-face-to-face practice training.

“I think I just carried out an absurd practice with a dummy on the screen. Unsurprisingly, I received feedback from the students that it was very disappointing” (participant 10).

“In the case of psychiatric nursing practice, when it was conducted online, students often said that it was difficult to catch non-verbal parts because most of them only heard verbal ones. Students say that they do not know what to say when they meet an actual mentally ill person, even after the practice” (participant 8).

### Acceptance of changes

Participants suffered helplessness and uncertainty about the effects of alternative practice education within the infection control system when they started involuntary alternative practice operations; however, they simultaneously reconsidered traditional practice methods. As the alternative practice operation was extended because of the prolonged pandemic situation, it was expressed as a change that must be accepted by recognizing the limitations of traditional practice methods as well as confirming the possibility of a safe educational environment and new practice training methods for infectious diseases.

#### Reconsideration of traditional practice methods

Participants encountered students accustomed to alternative practices and felt the need for flexible practices tailored to the subject characteristics. The traditional practice method with standardized uniformity and physical limitations in all practice subjects was reconsidered.

“If you look at the situation before COVID-19, especially in children's hospitals, when students go out for clinical practice and just observe, besides measuring the patient's vital sign. After two weeks of observation, the contents of the clinical practice manual were very limited, and there were cases where the same content was repeated and observed, and it was very boring for two weeks” (participant 16).

“Clinical practice is the best, but I think all professors and students complain about the limitations of clinical practice. If so, I think it is right that the direction of practice should change” (participant 1).

#### Confirmation of the feasibility of new practice training methods

Participants have continued to operate the practice in a new way, different from the existing face-to-face practice, through a long-term pandemic, and have accumulated know-how in the operation of the practice by finding technical support from the school and various contents available. In particular, it was confirmed that students' confidence and interest increased in an educational environment, considering that non-face-to-face online practice is safe from infectious diseases and repetitive learning is possible, confirming the possibility of a new practical education method.

“Communication, which is the core competency of nursing, can be sufficiently taught using VOD, ZOOM, and SP. It was similar to training SP and having a 1:1 conversation with students in ZOOM… I actually tried this method and it worked great” (participant 1).

“I changed the teaching method by using VR simulation and blending in a variety of contents, and I think the students had a lot of fun through various experiences” (participant 7).

“The first semester was chaotic, but after a while, I think it was an opportunity to run a wide range of practice classes with various cases to suit the situation” (participant 15).

### Preparation for practice training in the future

Participants felt the need for an educational method to freely operate in practice even during a pandemic. Accordingly, they argued that digital native learners and technology-based practice education, suitable for the new normal era, should be developed. To cope with the changing practice environment, it was suggested that future practice education should be prepared, including the exchange of new information, clinical advice through industry-academic linkage, and the establishment of a network for developing educational content.

#### Development of technology-based curriculum for practice

Participants reported that most of the students were digital natives called Generation Z, and thus, they were faster in accepting advanced technologies such as AR and VR than their instructors. One participant mentioned that practical education suitable for different learner characteristics should be developed and that even if the pandemic situation ended, it would not be possible to return to the earlier practice operation method completely.

“Even if the coronavirus pandemic is over, it will inevitably become a new normal era in which we cannot go back to the past. Because our students are of the MZ generation and are familiar with YouTube” (participant 12).

“When comparing before and after the pandemic, methods other than face-to-face should be strengthened for students to practice independently. As the pandemic exploded, the cost of VR development, wireless usage fees, and license fees decreased significantly. As long as there are wireless goggles, core skills are being developed to be used anywhere” (participant 1).

#### Responding to the changing practice training environment

Participants argued that industry-university cooperation and clinical advice are essential for exchanging information on new practice methods and creating a laboratory environment similar to clinical practice as preparation for future education. In addition, a network for the development of educational content should be established to respond to the changing practical educational environment.

“We need to create an environment that is most similar to clinical practice through the development of various modules, and it is best to seek advice from clinical teachers or clinicians for this part. Because so many things have changed in the past and now, I think industry-university cooperation is very important” (participant 1).

“It is my greatest wish to share information so that various teaching methods can be researched and utilized” (participant 9).

## Discussion

This study explored the laboratory and clinical teaching experiences of nursing professors in the COVID-19 pandemic era. As a result of analyzing the contents of the focus group interviews with nursing professors, four themes were derived: feeling helpless in the infection management system, uncertainty about the effectiveness of alternative practice training, acceptance of changes, and preparation for future practice training.

First, participants felt helpless in the infection management system. Under the infection management guidelines that changed with the spread of COVID-19, participants experienced unpredictable situations where laboratory and clinical education were stopped due to confirmed cases on campus. This is similar to other nursing colleges worldwide that have been affected by COVID-19 and have undergone clinical discontinuation (2, 32, 33). Regulations on credit completion at universities in Korea are strictly applied, and the criteria for credit and professors' approval are set for practical courses (19). After clinical practice was suddenly suspended, the professors had to ensure the prevention of infectious diseases and divide the class into a minimum number of people and operate laboratory and online classes. Participants reported burnout as a result of overwork, such as preparing educational materials to be recognized as clinical practice and increasing class hours, and as this situation continued, they gave up.

Burnouts cost both individuals and educational institutions because they undermine the function of teachers and cause poor quality education and health (34–36). Teachers experience a high level of work stress, especially university professors (22). More specifically, university professors are responsible for the essential task of training students in a variety of advanced specialized skills and promoting the development of science, technology, and social progress. In addition, university teachers must constantly interact with students, maintain a high level of professional performance, and meet targets and deadlines, even during a pandemic (37).

Based on the findings of the present study, it is necessary to reduce the psychological impact of the pandemic, as well as to improve and avoid these stressful situations among professors. It is suggested that supervising institutions take steps to encourage these practices and alleviate the problem of stress (22, 37, 38). Furthermore, it would be important for them to receive support in the form of additional educators and resources, as well as emotional support by introducing workshops or programs to strengthen their emotional resources (37, 38). In this way, the emotional environment in universities could be improved and the mental health of professors could be protected.

The second theme was that participants felt uncertain about the effects of the alternative practice training. As non-face-to-face education was conducted in an unexpected situation, it was taught without deep concern for teaching design principles or appropriate non-face-to-face educational tools (39). For professors to manage non-face-to-face practice, it is necessary to have support from educational institutions, including educational facilities, teaching design, technology, image storage ability, and a stable Internet connection (6, 40, 41). However, participants had to run alternative practice training without sufficient educational resources, such as school support, educational content, faculty members, and educational methods.

Planning for meaningful practice training is difficult and, in some cases, presents an ethical dilemma (16). Professors should choose the best teaching method and provide education to students (2, 3, 17). Participants said that alternative training is not equivalent to clinical practice and that they felt guilty about students graduating without clinical practice. As the pandemic is prolonged, students are unable to observe patient care at clinical sites, resulting in a lack of opportunities to acquire problem-solving skills and communication skills (20, 33). This also limits opportunities to learn to cooperate with colleagues and other professionals (42). It has been found that the practice was not simply converted into non-face-to-face practice; specifically, a strategy for maintaining and improving the quality of practical training is needed (9, 25). Therefore, it is necessary to identify the advantages and disadvantages of non-face-to-face practice and prepare effective, non-face-to-face clinical practices.

Third, the participants felt that accepting the non-face-to-face practice warranted a change. Alternative teaching methods such as technology utilization, real-time online interactive classes, online practice content, video streaming, and online practice homework have been used (25). In addition, the information of various teaching strategies for laboratory and clinical classes has accumulated in a short time. For example, participants utilized the simulation using a standard patient through Zoom and various practical content such as the mobile augmented reality, virtual reality, and video on demand for laboratory and clinical practice. This allowed participants to reconsider traditional practice methods and confirm the feasibility of new practice training methods. This non-face-to-face practice was enabled by the development and spread of educational technology (10, 43). Since the coronavirus pandemic outbreak, educational technology has been actively used in the education sector to facilitate interaction in online classes and systematically manage learning processes (3, 7). Instructors should consider the role and value of education in coping with changes such as the willingness to introduce a new educational technology system as a clinical teaching strategy, the intention to reflect it in the future curriculum, prior knowledge of new technology, and the perception of barriers to technology use (43).

Having started as a temporary measure to improve the situation, online education has led to new discussions in the field of nursing education (9, 16, 33). It is the instructor's role to provide a new learning environment that can integrate these technologies into the curriculum, including clinical practice after the pandemic (44, 45). This effort will provide an opportunity to overcome the problems of traditional nursing education (16, 20).

Traditional universities should not become aliases for today's online universities, but rather capitalize on digital technologies while maintaining a strong link between research and teaching activities and the unique on-campus student experience (17, 45, 46). Furthermore, online universities with extensive experience designing and delivering online learning programs may collaborate with traditional universities (17). This is especially true in technoscientific fields, where face-to-face practical and research activities are crucial.

Finally, the participants needed preparation for future practice training. Currently, nursing college students are a “digital native” generation exposed to the digital environment from childhood (17, 47). Nursing educators should understand the thinking, interests, and communication methods of Z-generation learners and determine the most effective teaching methods (48–51). Therefore, future education should modify the instructor's role in motivating, guiding, and advising the learner to the latter's role in designing and performing learning (52).

To move to the new normal, an approach different from the existing educational paradigm is required. Given these social requirements, what was considered important decades ago might no longer be relevant in the current education framework (16). Unfamiliar with non-face-to-face lectures, professors are currently experiencing difficulties. However, this situation also represents an opportunity to develop and provide higher-quality classes in order to prepare for similar situations in the future (53). Education innovation requires education for learning systems that can systematically implement it and instructors who can freely utilize it (17, 45). A crisis always represents a possibility as well; therefore, it is critical to prepare for another crisis in the future by examining, in detail, education in the current climate (46).

To cope with these changes, it is necessary to develop high-quality educational content based on creative scenarios and actively utilize them in actual curricula, away from the fixed thinking of traditional educational systems (16, 20, 40). This study confirmed the possibility and direction of alternative practical training by examining the laboratory and clinical teaching experience of nursing professors during the pandemic.

### Limitation

This study has several limitations. First, it is difficult to generalize the study results because the participants were recruited from South Korea and were only interested in nursing studies. Furthermore, the focus group interview had a small sample size. This study could not consider the perspectives of professors from other nursing colleges around the world. Owing to subjective understanding, there may be errors and biases in the quality and quantity of experience; hence, the results should be interpreted carefully. Additionally, this study lacked a student perspective. The inclusion of students' voices could better convey the situation from a holistic perspective. It is recommended that any future study be conducted at multiple time points and strengthened considerably by including nursing professors from other countries and determining the similarities and differences between professors from nursing as well as other health occupations.

## Conclusions

This study explored the laboratory and clinical teaching experiences of nursing professors and reflected on practical operations during the pandemic, as well as discussed the prospects of practical education after the pandemic and the role and direction for nursing professors. During the pandemic, nursing laboratories and clinical teaching experienced chaos and change. However, when experimenting with new teaching methods in various forms, it became an opportunity to consider non-face-to-face lectures, online platforms, and technology use. It is difficult to accurately predict how nursing practice education will change after the pandemic; however, the use of advanced technology in learner-centered education that can be flexibly selected according to the learner's situation is most likely to change the practice environment. In accordance with such changes, the roles and attitudes of teachers and educational institutions also need to change. During the pandemic, both the necessity and possibility of conducting non-face-to-face education have been confirmed; thus, it is expected that classes using technology will be actively developed in nursing practice education. Nursing professors should reflect upon and evaluate challenges to prepare for post-pandemic practical education.

## Data availability statement

The raw data supporting the conclusions of this article will be made available by the authors, without undue reservation.

## Ethics statement

The studies involving human participants were reviewed and approved by the Institutional Review Board (IRB) of the Catholic university of Korea (No. 2021-1445-0001) reviewed and approved the study protocol. The patients/participants provided their written informed consent to participate in this study.

## Author contributions

J-HK and S-YC designed the study and conducted the interview. S-YC and SJ analyzed the data. All authors drafted and reviewed the manuscript.

## Funding

This work was supported by a Korea Research Foundation grant funded by the Korean Government [MOEHRD, Basic Research Promotion Fund; NRF-2021R1A2C2006327].

## Conflict of interest

The authors declare that the research was conducted in the absence of any commercial or financial relationships that could be construed as a potential conflict of interest.

## Publisher's note

All claims expressed in this article are solely those of the authors and do not necessarily represent those of their affiliated organizations, or those of the publisher, the editors and the reviewers. Any product that may be evaluated in this article, or claim that may be made by its manufacturer, is not guaranteed or endorsed by the publisher.
